# Estimating Time of Infection Using Prior Serological and Individual Information Can Greatly Improve Incidence Estimation of Human and Wildlife Infections

**DOI:** 10.1371/journal.pcbi.1004882

**Published:** 2016-05-13

**Authors:** Benny Borremans, Niel Hens, Philippe Beutels, Herwig Leirs, Jonas Reijniers

**Affiliations:** 1 Evolutionary Ecology Group, University of Antwerp, Antwerp, Belgium; 2 Centre for Health Economics Research & Modelling Infectious Diseases (CHERMID), Vaccine & Infectious Disease Institute (VAXINFECTIO), University of Antwerp, Antwerp, Belgium; 3 Interuniversity Institute for Biostatistics and Statistical Bioinformatics (I-BIOSTAT), Hasselt University, Diepenbeek, Belgium; 4 Department of Engineering Management, University of Antwerp, Antwerp, Belgium; Ecole Polytechnique Federale de Lausanne, SWITZERLAND

## Abstract

Diseases of humans and wildlife are typically tracked and studied through incidence, the number of new infections per time unit. Estimating incidence is not without difficulties, as asymptomatic infections, low sampling intervals and low sample sizes can introduce large estimation errors. After infection, biomarkers such as antibodies or pathogens often change predictably over time, and this temporal pattern can contain information about the time since infection that could improve incidence estimation. Antibody level and avidity have been used to estimate time since infection and to recreate incidence, but the errors on these estimates using currently existing methods are generally large. Using a semi-parametric model in a Bayesian framework, we introduce a method that allows the use of multiple sources of information (such as antibody level, pathogen presence in different organs, individual age, season) for estimating individual time since infection. When sufficient background data are available, this method can greatly improve incidence estimation, which we show using arenavirus infection in multimammate mice as a test case. The method performs well, especially compared to the situation in which seroconversion events between sampling sessions are the main data source. The possibility to implement several sources of information allows the use of data that are in many cases already available, which means that existing incidence data can be improved without the need for additional sampling efforts or laboratory assays.

## Author Summary

Human and wildlife diseases can be tracked by looking at incidence, which is the number of new infections per time unit (typically day, week or month). While theoretically this would only be a matter of counting the number of newly infected individuals, in reality these data are difficult to acquire due to limited sampling possibilities and undetectable cases. This means that a method must be used to estimate the real incidence using a limited amount of data. For many infections, the concentration and quality of antibodies changes predictably over time, which means that one could use the antibody level at any point in time to back-calculate how much time passed since the infection entered the body. Other information, such as the age of the individual, or the presence of the pathogen, can also help to estimate when an individual became infected. Improving on existing methods, we developed a method that allows the use of a wide range of information sources for estimating individual time since infection. Using arenavirus infection in mice, we show that this method works well when sufficient background data are available, and that it can greatly improve the estimation of incidence patterns.

This is a *PLOS Computational Biology* Methods paper.

## Introduction

Infection incidence (the number of new infections per time unit) is a basic epidemiological measure that describes the transmission of an infection through time. Because the exact time at which an individual acquired an infection is difficult to assess, time of symptom onset is often used as a proxy (e.g. [1]). When the time between the moment of infection and symptom onset (the incubation period) is predictable, this proxy will not bias results, but incidence estimation does become problematic with asymptomatic infection or when incubation periods vary unpredictably [2].

Another common problem for measuring incidence is the time resolution of data, as the temporal precision of incidence is directly related to that of data “sampling”. Ideally, each new infection is detected and recorded immediately, but in reality this is rarely possible and new cases are often recorded at irregular intervals and a low number of time points, resulting in suboptimal resolution incidence data [3, 4]. Even more importantly, when sampling intervals are larger than the duration of symptoms, a proportion of cases will be missed. This problem is especially common in the case of wildlife diseases, as natural populations are often sampled incompletely and at relatively large intervals [5]. In such cases, indirect measures of incidence that rely on evidence of past infection are needed.

The presence of specific antibodies indicates whether an individual has previously been infected, and the distribution of different antibody (Ab) types (e.g. IgG, IgM, IgA) can give a rough indication of how recently the individual was infected [6–9]. If individuals in a population are sampled repeatedly, a seroconversion event in between two sampling events provides further information about the time since infection. Aside from being present or not, Abs vary over time in quantity (titer) and quality (avidity). On the condition that this temporal variation is sufficiently constant and predictable within and between individuals, these antibody dynamic properties can be used for a more accurate estimation of the time since infection.

Avidity (Ab-antigen bond strength) tends to increase with time since infection, which means that it can in some cases be used to back-calculate the time since infection. But although this method is used routinely, e.g. for human cytomegalovirus [10, 11], its sensitivity is low, and it can only differentiate between “recent” or “old” (e.g. less or more than 90 days since infection for cytomegalovirus) infection events [6, 12].

Temporal dynamics of Ab levels can be another source of information about time since infection. In such cases a model must be created that describes the course of Ab levels (titers) over time since infection using known serological response data. This model is then used to back-calculate, given an Ab titer, the time since infection, which in turn can be used for incidence estimation. This has been done for pertussis [13, 14], HIV [15, 16] and Salmonella [17, 18].

While this method is promising, significant improvements are still possible in two main ways. A common, important limitation for developing good time since infection models is the lack of detailed information about individual Ab dynamics, which limits the explanatory power of such models as they must in that case be estimated using cross-sectional instead of individual data (e.g. [18]). Experimental challenge studies, in which the exact time since infection is known, would be needed to describe and model the within-individual Ab dynamics needed to calculate time since infection, but these are notoriously difficult to conduct [19]. A perhaps more feasible approach to improving time since infection models would be to make optimal use of all available sources of information on the course of infection. While changes in Ab presence/titer over time can contain much information on time since infection and are the most obvious input data, additional information is contained in parameters such as the presence/quantity of the pathogen (or of other immune response markers), individual age (e.g. for typical childhood infections, young individuals are more likely to have been infected recently than older ones) or season (e.g. for seasonal infections, individuals are more likely to have been infected recently during or short after the peak transmission season).

Here, we present a novel method that allows the integration of multiple serological biomarkers (Ab presence/absence/titer, pathogen presence/absence) as well as additional prior knowledge (e.g. age, season, capture probability) to inform a semi-parametric mixed model that back-calculates the time since infection of each individual, in a Bayesian framework. The integration of multiple sources of information ensures the optimal use of data that are often already available but not yet taken into account.

We apply this method to estimate the incidence of Morogoro virus (MORV) infection in Natal multimammate mice (*Mastomys natalensis*). This model system is used because the epidemiological and demographic parameters necessary for testing this method are well known for this infection. MORV is a member of the arenaviruses, a family of zoonotic viruses that includes viruses able to cause hemorrhagic fever in humans after acquiring infection from wild rodents (e.g. Lassa virus (LASV), Junin virus, Machupo virus) [20]. It is restricted to East-Africa, and while it does not seem to cause disease in humans it is closely related to Lassa virus which causes Lassa hemorrhagic fever in West-Africa, and with which it shares the same host species. Because both the population ecology of the rodent host *M. natalensis* and the infection ecology of MORV have been studied thoroughly (driven by the host’s status as an agricultural pest species and the virus’ close resemblance to LASV) [21, 22], MORV infection provides a good model system for testing the current method.

As is the case for other time since infection methods, two types of datasets are needed to estimate incidence. A first dataset, consisting of any type of data that contains information on the temporal course of infection (e.g. Ab titer dynamics in an infected individual), is used once in order to create an integrated model of individual time since infection. Once created, this model can be used to estimate incidence from cross-sectional sampling data that ideally (but not necessarily) includes repeated measures of individuals.

We use a wildlife disease model system to develop and test the method because detailed individual-level infection/antibody dynamics are available, but also to show that the method is applicable to both human and wildlife infections. Because it is usually difficult to monitor infections at a high time-resolution, this method can provide a way to improve the quality of longitudinal data without having to increase sampling efforts.

## Methods

In the following, we show how different types of data (e.g. levels, presence/absence) can be used to estimate the time of infection, and as a proof of principle we apply the method to MORV transmission in the multimammate mouse *M. natalensis*. For each type of data we present a generalised method and immediately apply it to MORV, and we show how to use individual estimates of the time of infection to estimate incidence in the population. Finally, through the use of simulated MORV transmission data we investigate method performance under different conditions.

**Fig 1:**
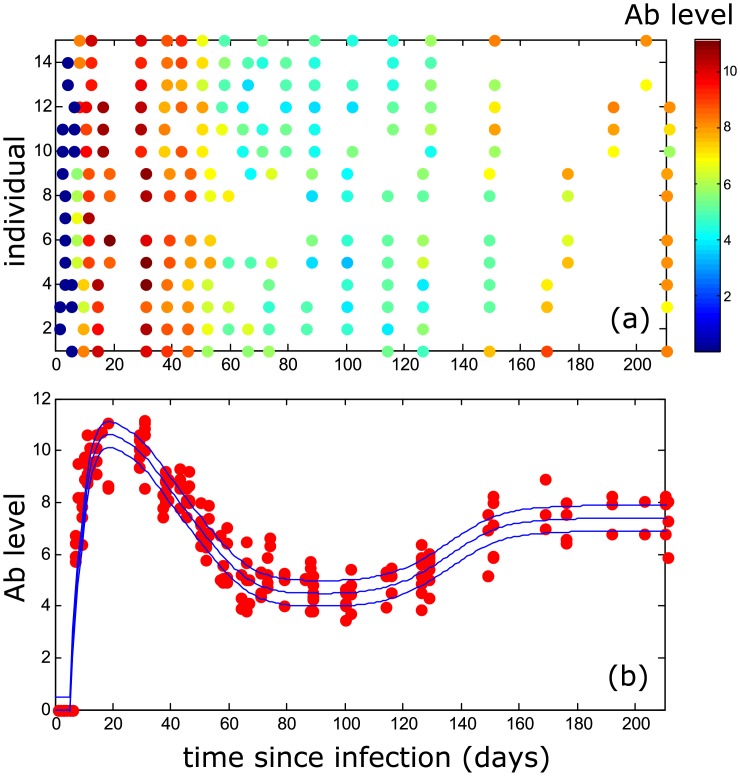
Temporal variation of antibody levels obtained from experimental data [23] for 15 different individuals (a) and for all individuals combined (red dots) with fitted function mean and standard deviation (blue lines) (b).

MORV Ab level dynamics and virus presence in blood and excretions (urine, feces, saliva) have been quantified previously in a challenge study, described in [23], where multimammate mice from a breeding colony were injected with cultured MORV and sampled frequently for 210 days, which is more than their average lifetime in natural conditions (Fig 1 and [23]).

### Back-Calculation Model

#### Bayes’ rule

In the following, we assume that an individual can be encountered at different times, at which it can be tested for different types of information: Ab level, pathogen presence, age, body weight, sex, etc. For each measurement type *k*, the experimental information for a single individual can be represented by a vector $X^{k}=\left[x_{1}^{k},x_{2}^{k},...,x_{n}^{k}\right]$, of which the different coordinates represent the responses that have been measured at times ***T*** = [*t*_1_, *t*_2_, …, *t*_*n*_] for a particular individual.

Decoding the information about the individual time of infection *θ* from these experimental data ***X***^***k***^ essentially comes down to the calculation of *P*(*θ*|***X***^***k***^, ***T***), which is the probability that, given the information ***X***^***k***^ measured at times ***T***, the tested individual was infected at time *θ*. In order to calculate *P*(*θ*|***X***^***k***^(***T***), ***T***), we make use of Bayes’ Rule to arrive at

$$\begin{array}{c} P\left(\theta |X^{k},T\right)=\frac{P(T)}{P(X^{k},T)}P\left(X^{k}|T,\theta \right)P\left(\theta |T\right). \end{array}$$ (1)

Both the numerator and denominator of the first factor are independent of *θ*, and consequently this fraction can be inferred from the fact that ∫*P*(*θ*|***X***^***k***^, ***T***)*dt* = 1. Calculating the posterior probability *P*(*θ*|***X***^***k***^, ***T***) is then reduced to the calculation of *P*(***X***^***k***^|***T***, *θ*), i.e. the likelihood that a time of infection *θ* produces the information ***X***^***k***^ at times ***T***, and *P*(*θ*|***T***), i.e. the prior for *θ* if we assume that the individual was encountered at times ***T***. In the following, we describe how to model *P*(***X***^***k***^|***T***, *θ*) and *P*(*θ*|***T***) using different sources of information.

### Modeling *P*(***X***^***k***^|***T***, *θ*)

The estimation of the time of infection *θ* can be based on different dimensions of the immune response that each require a slightly different approach. In the following we consider two different sources of information.

#### Using level information

In a situation where the level of a measured biomarker (e.g. Ab or pathogen levels in blood) exhibits predictable temporal variation we can extract information on the time since infection from the measured level [18]. For example, in the particular case of MORV, Fig 1 clearly shows that the Ab-level contains information about the time since infection.

First, let us consider the case of a single level $x_{i}^{k}$ where we have to determine $P(x_{i}^{k}|t_{i,}\theta )$, i.e. the conditional probability of measuring level $x_{i}^{k}$ if the individual was infected at time *θ* and tested at time *t*_*i*_. As is clear from the data shown in Fig 1, a particular value of the time since infection *t*_*i*_ − *θ* does not necessarily result in a single possible biomarker level due to variation caused by inherent measurement errors, temporal variation and/or individual differences. The measured level $x_{i}^{k}$ at time *t*_*i*_ can be written as

$$\begin{array}{ccc} x_{i}^{k} & = & L\left(t_{i}-\theta \right)+\delta_{i}, \end{array}$$ (2)

$$\begin{array}{ccc} \delta_{i} & ∼ & N(0,\sigma \left(t_{i}-\theta \right)), \end{array}$$ (3)

i.e. the mean level corresponding to a time since infection, *L*(*t*_*i*_ − *θ*), plus an ‘error’ *δ*_*i*_. This model and the error distribution are system-specific, and can take any empirical form as long as it adequately describes the course of the biomarker over time. It is typically derived from experimental infection data. Here, we assume that the error is normally distributed, with a variance *σ* that may be dependent on the time since infection *t*_*i*_ − *θ*, because this is probably a common situation. Using these approximations, we arrive at the following conditional probability for a single level measurement:

$$\begin{array}{c} P\left(x_{i}^{k}|t_{i,}\theta \right)=\frac{1}{\sqrt{2\pi }\sigma \left(t_{i}-\theta \right)}\exp\left\{-\frac{1}{2{\left[\sigma \left(t_{i}-\theta \right)\right]}^{2}}{\left[x_{i}^{k}-L\left(t_{i}-\theta \right)\right]}^{2}\right\}, \end{array}$$ (4)

with *t*_*i*_ − *θ* the time since infection.

This model describes the conditional probability based on a single measurement, but one often has more information on the evolution of the levels, since an individual may be encountered and tested at different times. In this case, the temporal level information is contained within a vector ***X***^***k***^ of which the different coordinates represent the responses measured at times ***T***. If we again consider the individual to have been infected at time *t*, Eqs 2 and 3 can be generalized to

$$\begin{array}{ccc} X^{k} & = & L(T-\theta )+\delta , \end{array}$$ (5)

$$\begin{array}{ccc} \delta & ∼ & N(0,\Sigma ), \end{array}$$ (6)

with the covariance matrix **Σ** over all *n* times the individual was tested, and *δ* a *n* − dimensional vector drawn from a multivariate normal distribution. Finally, this results in

$$\begin{array}{l} P(X^{k}|T,\theta )\;=\;\frac{1}{{\left(2\pi \right)}^{n/2}{\left|\Sigma \right|}^{1/2}}\\ \;\times \text{exp}\left\{-\frac{1}{2}{\left[X^{k}-L\left(T-\theta \right)\right]}^{T}\Sigma^{-1}\left[X^{k}-L\left(T-\theta \right)\right]\right\}. \end{array}$$

The covariance matrix would typically be inferred from experimental data and accounts for the possible interdependence of level responses at different times. Indeed, the error ***δ*** of different measurements may not be independent over time. Also, it is possible that part of the variance is caused by individual differences, i.e., ***δ*** = ***δ***_ind_ + ***δ***_noise_, as some individuals may have a stronger immune response (higher overall levels) than others.

#### Applied to MORV: Ab level

We apply this to MORV by considering information about one Ab (IgG) measurement, shown in Fig 1. First, in order to arrive at errors that can be adequately described by a normal distribution, we take the logarithm of the Ab level. Next, we estimate *L*(*t*) by fitting a smooth spline to the data to arrive at the curve shown in Fig 1b.

Then, we subtract the corresponding *L*-value from each datapoint and calculate to what extent individual variation and temporal variation account for the variance observed in the residual errors, as this would then have to be taken into account in the covariance matrix. Using an ANOVA, we found no significant effect of individual (p = 0.085) or time (p = 0.089) on the variation of the residual errors. Based on the sum of squares, the relative contributions to the total variance were estimated to be 1.3% for time and 9.6% for individual. From this analysis, we find that the effects of individual and time can be ignored, compared to the residual variance, and consequently we consider the covariance matrix to be proportional to the unitary matrix, *σ*^2^ ***I***, independent of *t*. All off-diagonal elements are assumed zero. The residual standard deviation was measured to be *σ* = 0.99 and approximated to 1.

Note that although we here estimate *L*(*t*) using a spline method and with the assumption that there is no individual or temporal effect on variation, *P*(***X***^*k*^|***T***, *θ*) can be modeled using any method, as long as the model adequately describes the data. Indeed, an alternative to using a spline method is to use a mechanistic model, and an alternative to determine the appropriate covariance structure is to use a hierarchical modelling approach in which likelihood theory is used to test the contribution of the different sources of variability (see e.g. [17, 18, 24]).

#### Using presence/absence information

Often, information on presence/absence of biomarkers is more easily available than level data. This can be due to biomarker assay limitations, because level variability of the measured biomarker is too high and unpredictable, or because the levels do not change sufficiently over time. In such situations, it may be possible to use presence ($x_{i}^{k}=1$) or absence ($x_{i}^{k}=0$) of a biomarker (e.g. IgG, IgM, virus), often measured using assays that result in values above or below a detection threshold. Given that an individual was infected at time *θ*, the probability of biomarker presence or absence $x_{i}^{k}$ at time *t*_*i*_ is given by

$$\begin{array}{c} P\left(x_{i}^{k}|t_{i},\theta \right)=x_{i}^{k}\left[2p(t_{i}-\theta )-1\right]+\left[1-p\left(t_{i}-\theta \right)\right], \end{array}$$

which would typically be derived from experimental infection data.

In the case of multiple (*n*) measurements, presence/absence data are contained in a vector ***X***^***k***^, with *n* measurements $\left[x_{1}^{k},x_{2}^{k},...,x_{n}^{k}\right]$, where $x_{n}^{k}$ is the *n*-th measurement indicating presence (1) or absence (0). Assuming that measurements at different times are independent, we can write

$$\begin{array}{c} P\left(X^{k}|T,\theta \right)=\prod_{i=1}^{n}P\left(x_{i}^{k}|t_{i},\theta \right). \end{array}$$ (7)

#### Applied to MORV: Ab presence

Usually in epidemiology only information about Ab presence or absence (seroconversion events) is used to estimate the time since infection, resulting in incidence estimates with low temporal resolution [25, 26]. Here, we use that situation as a reference, in order to evaluate the improvements offered by using Ab level instead of only presence/absence data.

When only considering Ab presence, the measurement $x_{i}^{ab}$ is a binary variable of which the value depends on whether Ab was present (1) or absent (0) at time *t*_*i*_. The probability *p*^*ab*^(*t*) of detecting Ab in blood if an animal was infected at *t* = 0 is then given by

$$\begin{array}{c} \begin{array}{l} p^{ab}\left(t\leq 6\right)=0\\ p^{ab}(t>6)=1, \end{array} \end{array}$$

as it was found that Ab are never present before day 7 after infection [23]. After this initial period, we assume the test to be sensitive enough to detect Ab presence with a probability of 1 (Fig 1).

#### Applied to MORV: Virus presence in blood

Based on experimental data, the probability *p*^*vb*^(*t*) to detect virus in blood (Vb) if an animal was infected at *t* = 0 can be adequately modeled by

$$\begin{array}{c} p^{vb}\left(t\leq 1\right)=0\\ p^{vb}(1<t\leq 8)=1\\ p^{vb}(t>8)=\text{exp}\left[-0.3\left(t-8\right)\right], \end{array}$$

as shown in Fig 2a.

**Fig 2:**
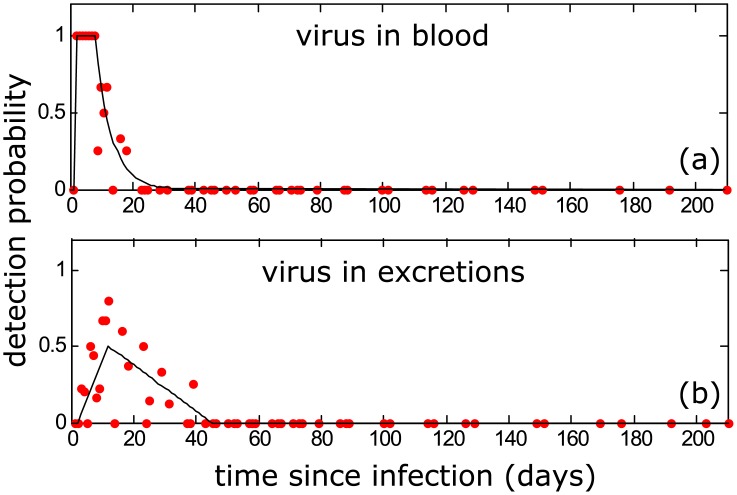
Probability of virus presence in blood (a) and excretions (b), estimated from experimental data [23]. Detection probability is given by the proportion of tested individuals that was RNA-positive on a given sampling day.

#### Applied to MORV: Virus presence in excretions

Similar to using information on Vb, another source of information is the presence/absence of virus in excretions (Ve; urine, saliva or feces). Based on the experimental data shown in Fig 2b, we model the probability *p*^*ve*^(*t*) to detect Ve if an animal was infected at *t* = 0 as

$$\begin{array}{c} p^{ve}\left(t\leq 2\right)=0\\ p^{ve}(2<t\leq 12)=\left(t-2\right)/20\\ p^{ve}(12<t\leq 45)=\left(45-t\right)/66\\ p^{ve}(t>45)=0. \end{array}$$

#### Combined biomarker information

After modeling all biomarkers of interest, the separate models can easily be combined into one conditional probability of the time of infection that incorporates information about different biomarkers, including levels (or presence/absence) of different antibodies (e.g. IgG, IgM, …), pathogen (e.g. virus, bacteria) concentration (or presence/absence), and in different tissues (blood, excretions, organs, …). One should keep in mind that the errors, levels or presence of some of the different sources can be correlated, which should be taken into account in the covariance matrix.

If we assume *N* independent sources of information, we can combine these by simple multiplication of their respective conditional probabilities to arrive at

$$\begin{array}{c} P\left(X|T,\theta \right)=\prod_{k=1}^{N}P\left(X^{k}|T,\theta \right), \end{array}$$ (8)

where *k* runs over *N* different sources of information. The resulting conditional probability can then be inserted into Eq 1.

### Modeling *P*(*θ*|***T***)

Because an individual can of course only have been infected when it was alive and present in the population, the estimation of *θ* can be improved by incorporating prior information about the probability of an individual being alive/present, i.e. by modeling *P*(*θ*|***T***). Here, we show how to implement information on mortality rate and maximum life span, age at the time of sampling, and encounter probability, but note that any source of information can be used in a similar way as long as it results in a realistic prior distribution.

**Fig 3:**
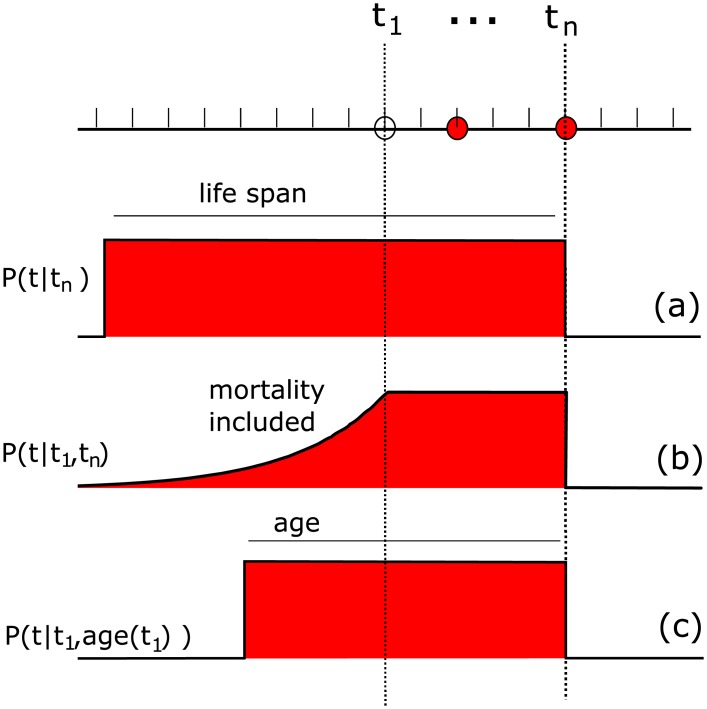
Example of the possible use of information about maximum life span (a), mortality rate (b) and individual age (c). The three dots on the time axis indicate the different times at which a hypothetical individual was sampled. The red blocks indicate the probability of being alive at a certain point back in time, which can be included as prior information on the estimated time of infection.

Knowledge about the maximum life span can be informative because it sets an upper boundary to the possible time since infection, and is especially useful in situations where the maximum life span is short relative to the possible time since infection. If an individual was last tested at time *t*_*n*_ and the maximum life span is known, then the prior distribution *P*(*θ*|***T***) can be reduced to

$$\begin{array}{c} P\left(\theta |T\right)∼\frac{1}{\text{life}\;\text{span}}\left[\theta >(t_{n}-\text{life}\;\text{span)}\right]\left[\theta <t_{n}\right], \end{array}$$

with [. < .] is a boolean operator that returns 1 or 0 when the equality is true or false, as shown in Fig 3a.

Similarly, one could make use of the mortality rate, as this is directly associated with the possible age of an individual. If an individual was first encountered at time ***t***_1_ and we assume a mortality rate *γ* as inferred from data, we arrive at prior distribution

$$\begin{array}{c} P\left(\theta |T\right)∼\max\left[\exp(\gamma \left(\theta -t_{1}\right)),1\right]\left[\theta <t_{n}\right], \end{array}$$

as shown in Fig 3b. This figure clearly shows that, due to mortality, it becomes increasingly unlikely for individuals to have been alive, and therefore infected, further in the past.

When more precise information exists on the age of an individual focus individual (which is trivial for humans, while for wild animals this can be based on physiological or morphological features such as weight), this can be taken into account explicitly by including

$$\begin{array}{c} P\left(\theta |T\right)∼\left[\theta >(t_{1}-\text{age}\left(t_{1}\right))\right]\left[\theta <t_{n}\right], \end{array}$$

if the individual was first encountered at time *t*_1_, see Fig 3c.

More applicable to wildlife infections is the use of encounter probability (typically termed trapping or capture probability, but for consistency and human application we will here refer to it as encounter probability). In a typical capture-mark-recapture study, only a proportion of individual is captured during each session, and well-developed methods exist for estimating encounter probability [27, 28]. This encounter probability can be used to estimate the likelihood of an individual being alive at a certain point in time, assuming a closed population during that time (no migration). If an individual is first encountered at time *t*_1_, the probability of it being born at time *θ* decreases with *t*_1_ − *θ*, as it becomes increasingly unlikely that it was not encountered during (*t*_1_ − *θ*) / Δ*t* trapping sessions.

If we estimate encounter probability *p*_*enc*_ for every trapping session, this information can be used to further improve the prior time distribution:

$$\begin{array}{l} P(\theta |T)\;∼\text{ max}\left[{\left(1-p_{enc}\right)}^{(t_{1}-\theta )/\Delta t},1\right]\left[\theta <t_{n}\right]\\ \;\approx \text{ max}\left\{\text{exp}\left[p_{enc}\frac{\left(\theta -t_{1}\right)}{\Delta t}\right],1\right\}\left[\theta <t_{n}\right], \end{array}$$

where Δ*t* is the sampling or trapping interval time, and with the latter approximation valid only when *p*_*enc*_ < <1. This approach only holds if one can assume a closed population where every individual was in the population during its lifetime and the effects of migration are negligible.

One could also use seasonal information or cross-sectional data to inform the prior *P*(*θ*|***T***), or in fact any other data source that contains any type of information about the time since infection.

### Decision Criterion

Given the resulting posterior probability *P*(*θ*|***X***, ***T***), the observer still has to use a decision criterion to decide which time of infection *θ* is most likely. Probably the most obvious decision criterion is the mean squared error (MSE) of the time since infection by selecting the ${\hat{\theta }}_{i}$ for which

$$\begin{array}{c} MSE=\frac{1}{N_{ind}}\sum_{1=1}^{N_{ind}}{\left[\hat{\theta }\left(i\right)-\theta \left(i\right)\right]}^{2}, \end{array}$$

with *i* running over a population of *N*_*ind*_ individuals, is minimal. It can be shown that this is the case for $\hat{\theta }=\int d\theta P\left(\theta |T,X\right)\theta$ [29].

In order to assess the quality of the estimates, the remaining uncertainty on the time since infection can be inspected conditional on the observed data (***X***, ***T***), which can be quantified using the conditional entropy *E*(*θ*|***X***, ***T***) [29], i.e.,

$$\begin{array}{c} E\left(\theta |X,T\right)=\int_{\theta^{\prime }}d\theta^{\prime }p\left(\theta^{\prime }|X,T\right)\log_{2}p\left(\theta^{\prime }|X,T\right), \end{array}$$

where *θ*′ runs over all possible time since infection values. Conditional entropy is a commonly used measure in information theory that quantifies (in *bits*) the remaining amount of uncertainty about the actual value of the quantity of interest (here: time since infection). The highest entropy is attained for a uniform posterior probability distribution (maximum uncertainty), whereas the minimum (zero) entropy is obtained when there is no uncertainty left about the actual value [29]. In an epidemiological context, the entropy value can be used to improve the reliability of estimated incidence (see next paragraph) by removing all estimates of *θ* for which the entropy value is larger than a threshold value. The choice of this threshold value will mostly depend on the trade-off between sample size and estimation error: a low threshold value will generally result in a higher quality of the remaining *θ* estimates, but at the cost of reducing the final size of the dataset, and will therefore be dataset-specific.

### Estimating Incidence

One of the main purposes of knowing the time of infection of an individual is to analyse and model infection incidence on a population level. To this end, we need to estimate the time of infection *θ*_*i*_ for all sampled individuals *i* in the population and count the number of newly infecteds on a regular (usually daily) basis. Because in most situations only a proportion of individuals will be encountered and sampled, the “real” proportion of new infections needs to be estimated. This can be done by dividing the number of infecteds by an estimate of the proportion of encountered individuals. Given a certain sampling interval Δ*t* and an encounter probability at each session (*p*_*enc*_), this proportion can be approximated by

$$\begin{array}{c} \text{proportion}\;\text{encountered}=\gamma \int dt\exp\left(-\gamma t\right)\left[1-{\left(1-p_{enc}\right)}^{t/\Delta t}\right], \end{array}$$

where the integral runs over all the survival times *t* following an exponential distribution with 1/*γ* (the average lifespan of an individual in our simulation), *t* / Δ*t* is the approximate number of sampling sessions during lifetime *t*, and (1 − *p*_*enc*_)^*t*/ Δ*t*^ is the approximate probability that an individual is never encountered during these sessions.

### Application to MORV Infection in *M. natalensis*

Next, in order to test the back-calculation scheme, we need a dataset of individuals in a population, with full knowledge of their infectious status at each moment. Also, to test the efficacy of the method as a function of sample size (with regard to intervals between sampling sessions as well as the sampling effort), we need datasets collected under different trapping regimes. We therefore simulate MORV transmission in a population of multimammate mice, “sampled” in different trapping sessions, with each individual given simulated infection attribute data based on the experimentally-derived [23] course of Ab levels and probability of virus presence in blood and excretions. These simulated data are equivalent to epidemiological data obtained through surveys with repeated sampling, but now of course with the difference that our simulated data are completely known for testing purposes. All simulated data, as well as the Matlab code used to apply the time of infection estimation method, can be found in S1 Data.

As input for the model, we use simulated data from an existing individual-based spatially-explicit SEIR model, which models the population dynamics and the transmission of Morogoro virus in *M. natalensis* [30]. In this model, individuals are born in the susceptible (S) state and can become infected through contact with infectious (I—infectious state) individuals. When infected, they enter a latent stage (E—exposed state) during which they cannot transmit the virus, until they become infectious (I) after around 6 days. After around 45 days they stop being infectious, recover from the infection (R—recovered state) and remain immune against re-infection for the remainder of their life. Latent and infectious periods were simulated assuming an exponential distribution. The simulation is run over a total area of 10ha, but in order to recreate a realistic situation in which individuals can move freely in and out of the study site, only the individuals that are encountered within a central 5ha area were available for “trapping”. Realistic population densities and fluctuations are used, ranging between around 10 and 150 per ha. After a simulation burn-in period, two years of data are considered (from day 1000 until 1730).

Throughout the simulation we keep track of each individual’s age, time since infection *t*, and we simulate trapping sessions with a time interval Δ*t*, in which every individual present in the 5ha area has a probability *p*_*trap*_ to be trapped. Whether an individual is trapped or not is determined using pseudo random numbers. This way, for every individual we can generate an artificial set of measurements (***T***, ***X***^***k***^) that we can then use to estimate the time of infection $\hat{\theta }$. ***X***^*ab*^ are random realisations according to the multivariate distribution shown in Eq 8 at times ***T***. ***X***^*vb*^ and ***X***^*ve*^ are random draws with respective probabilities *p*^*vb*^ and *p*^*ve*^ at times ***T***. We vary the time intervals between capture sessions using Δ*t* = 1, 7, 14, 28, 56 days, as well as the probability for each of the individuals to be captured using *p*_*trap*_ ∈ (0, 1).

We implement a maximum life span of *M. natalensis* of 450 days based on [31]. The average mortality rate (averaged across the year) is calculated from the simulation data, and estimated to be *μ* = 0.008537 mice/day (average life span of 117 days). Both maximum and average life span are used as prior information for all time of infection estimates.

## Results and Discussion

### Ab Level vs Presence/Absence, without Additional Information

**Fig 4:**
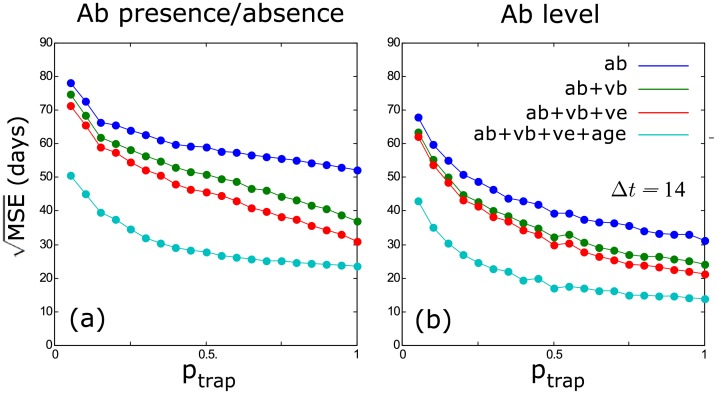
Estimation error ($\sqrt{\text{MSE}}$) on the time of infection for different encounter probabilities (p_trap_) and for different levels of included prior information (ab: antibody, vb: virus in blood, ve: virus in excretions, age: individual age); (a) is based on antibody presence/absence, while (b) is based on antibody levels. The trapping interval was 14 days for all situations.

**Fig 5:**
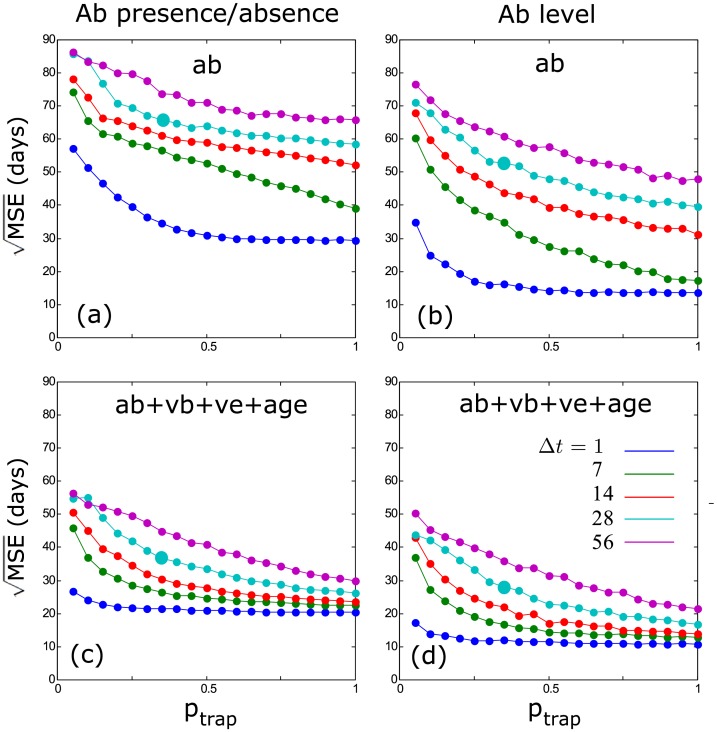
Estimation error ($\sqrt{\text{MSE}}$) on the time of infection for different encounter probabilities (p_trap_) and different sampling intervals (Δ*t*) (ab: antibody, vb: virus in blood, ve: virus in excretions, age: individual age); (a) and (c) are based on antibody presence/absence, while (b) and (d) are based on antibody levels; (a) and (b) only include antibody information, while (c) and (d) include all available information. The larger dots on the 28 day line indicate the situation for which incidence plots are shown in Fig 6.

**Fig 6:**
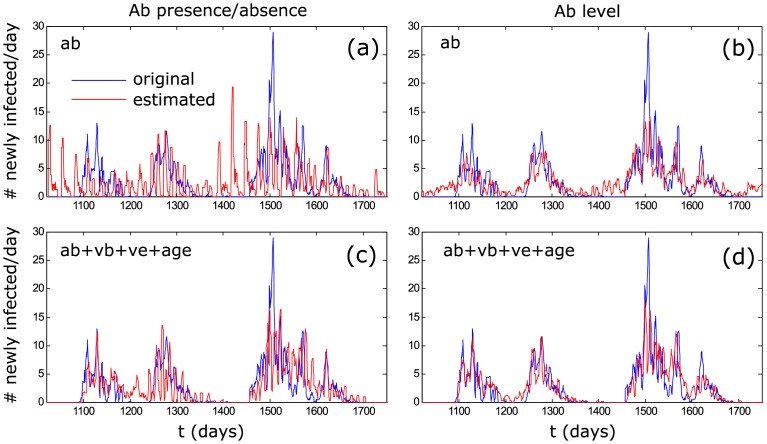
Simulated (blue) and estimated (red) incidence using different sources of information; (a) and (c) are based on antibody presence/absence, while (b) and (d) are based on antibody levels; (a) and (b) only include antibody information, while (c) and (d) include all available information; (a) represents the situation that is mostly used in existing studies. Larger dots on Fig 5 (28 day line) indicate the situation for which incidence plots are shown.

The estimation of the time since infection is much improved by the use of Ab levels, as opposed to when only using Ab presence/absence data (Figs 4 and 5). The use of Ab levels also results in a much better reconstruction of incidence dynamics, even without including additional information such as virus presence or individual age (Fig 6). When using Ab presence/absence data, incidence can only be estimated with a low temporal resolution, the main consequence being that the peaks and troughs of the incidence dynamics were estimated badly (Fig 6). Although the incidence peaks are estimated quite well when using Ab levels, the periods of low incidence are still often over-estimated (Fig 6).

### Including Additional Information

The inclusion of additional information (Vb, Ve, individual age) greatly improves the estimation of time since infection and incidence (Figs 4–6). Interestingly, this effect is more pronounced when using Ab presence/absence than when using Ab levels. The combined use of Ab levels and other available information results in the highest quality reconstruction of incidence dynamics, where the inclusion of additional information mainly reduces the previously observed over-estimation of low incidence levels between peaks.

Nevertheless, even when using Ab presence/absence instead of Ab level data, incidence can be reconstructed well when including Vb, Ve and individual age. This is encouraging, given the fact that many datasets, especially for wildlife infections, already contain some or all of this information; it means that by applying the back-calculation method, many existing incidence estimations can be improved significantly without additional laboratory or sampling efforts.

### Sampling Frequency and Encounter Probability

The quality of the estimates strongly depends on sampling frequency (or trapping interval) and the proportion of individuals that is encountered (or trapped) and sampled. While more additional prior information always results in a better estimation of the time since infection, we see that, at low (realistic) encounter probabilities, this effect is strongest (Fig 4). We also observe that a higher sampling frequency results in better estimates (Fig 5), and this is largely an effect of increased sample sizes: when adjusting the trapping probability to equalise sample sizes of different sampling frequencies, this effect mostly disappears (S1 Fig). This means that, in theory, similar results can be reached for any sampling frequency or trapping interval, but only if the sampling effort is increased so that a sufficient number of individuals can be sampled. Nevertheless, we observe that long sampling intervals (28–56 days) generally result in lower quality estimates (S1 Fig), indicating that a shorter interval would still be preferred.

### Entropy Threshold

**Fig 7:**
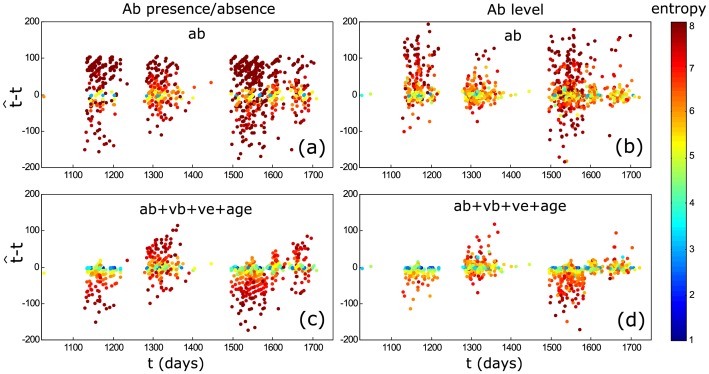
Difference between the estimated and real time since infection in relation to the entropy level (bits). Each datapoint is a “sampled” individual.

In the model, we introduce the use of entropy (which is inversely related to information) as an indicator of the amount of uncertainty contained by an estimate. Fig 7 shows how estimates of the time since infection with a higher deviation from the real time since infection generally also contain less information (i.e. have a higher entropy). Similarly, we observe a strongly positive correlation between the MSE of the estimated time of infection and the entropy level (S2 Fig). Therefore, by removing estimates above a critical entropy value, the MSE can be lowered, albeit at the cost of a lower sample size. Because of this trade-off it is not possible to suggest an optimal critical entropy cut-off value, which should rather be chosen depending on the specific situation, sample size and quality of available information.

### Model Limitations

Although the model performs well and seems promising for a wide range of situations, there are a number of important assumptions and prerequisites that must be met before it is possible to apply the model to data. First, of course, empirical data on the dynamics of biomarkers (e.g. antibodies, viral RNA, etc) within individuals must be available. These can be relatively straightforward data such as knowledge about when after infection individuals seroconvert and how long antibodies remain detectable, or more elaborate information such as the temporal variation of antibody and virus levels after infection.

Then, these data can only be used if they are sufficiently consistent across individuals. If there is too much inter-individual variation in the shape of biomarker dynamics, it will not be possible to predict individual patterns. This does not however mean that there can not be individual variation in the magnitude of the response, as this would in fact be easy to implement into the model.

Further care must be taken if biomarker data have been obtained through laboratory experiments. Because laboratory conditions are often controlled and limited, natural variation in factors such as individual differences in immune response, stress, secondary infection, initial dose, boosting, etc. may result in different biomarker dynamics that could invalidate a time since infection model if they can not be incorporated into the model [32]. Ideally this is tested through a comparative study between laboratory and field patterns, but if such a study has not been done we must assume that the patterns observed in laboratory conditions apply to the natural situation.

Other factors that could render the use of a time since infection model difficult are the existence of maternal antibodies and the simultaneous presence of chronically and acutely infected individuals, as these factors would be difficult (but not necessarily impossible) to disentangle and take into account. On the other hand, under certain conditions these factors may even improve the model, as they provide additional information; for example, if maternal antibodies only occur for a certain period in newborn individuals, and if maternal antibodies can be distinguished from other antibodies (e.g. because of lower levels or using a different assay), this information can likely improve the estimation of the time since infection when incorporated into the model.

### Model Novelty and Applicability

Under the conditions described here, the model is a significant improvement on existing models (e.g. [14, 17, 18, 33]). It provides a relatively simple probabilistic framework for the incorporation of any data source that can inform the estimation of time since infection, such as biomarker level/presence, age, season, sex, weight, etc., and thus allows for the use of individual-level data to interpret cross-sectional survey data and estimate population-level incidence. An important strength of the method is that it does not assume a certain form for the underlying models, which makes it possible to use a general spline method but also a more specific ordinary differential equation (ODE) method when a good ODE can be found (e.g. [17]).

More specifically for wildlife infections, the method has the potential to enhance existing long-term data. Often, large logistical efforts are necessary to collect longitudinal data on wildlife infections, and even the best datasets have a relatively low temporal resolution, typically consisting of monthly (but often less frequent) capture sessions [5, 34–37]. Prevalence or incidence patterns resulting from such data are usually also limited to this capture frequency, and to our knowledge the only efforts for improving these data have been the rough estimation of seroconversion events between two capture sessions (e.g. [38, 39]). We have shown however that by integrating multiple sources of information (that have often already been collected or analysed), the quality of incidence data can be greatly improved, especially (but not uniquely) when predictable antibody level dynamics are available.

### Conclusion

Due to its flexibility, the model presented here allows the integration of multiple sources of information, thus making optimal use of all available data for estimating individual times of infection and population incidence. It provides a conceptually simple, flexible framework for estimating the time since infection and incidence of human as well as wildlife infections, and can potentially be used to significantly improve incidence estimation based on already existing data.

## Supporting Information

S1 FigEstimation error ($\sqrt{\text{MSE}}$) for different sampling intervals (Δ*t*) and different sample size corrected encounter probabilities (m) (ab: antibody, vb: virus in blood, ve: virus in excretions, age: individual age); (a) and (c) are based on ab presence/absence, while (b) and (d) are based on ab levels; (a) and (b) only include ab information, while (c) and (d) include all available information.In order to adjust the trapping probability so that the number of individuals captured during a month is more or less teh same, a constant *m* was used such that ***p***_***trap***_ = 28*m / **Δ*****t***, with ***m*** ∈ [1, 20]. Smaller ***m***-values correspond with a lower *p*_*trap*_ but a similar number of individuals.(EPS)

S2 Fig (a): Frequency distribution of entropy values for different levels of additional information; (b) Correlation between entropy and the mean absolute difference between estimated and real time since infection. Different lines (a, b, c, d) correspond with the respective situations in Fig 7 in the main text.
(EPS)

S1 Data Matlab code and transmission model simulation results. This file contains the Matlab code used to generate the results in this article, as well as the data. The datafile consists of 3 matrices, where the columns are the daily model situations (increasing in time from left to right, starting after a 500-day burn-in period and selected within a 5 ha grid as described in the methods) and the rows represent all individuals present in the simulation. Empty cells (no individuals) are indicated by a negative number. The id matrix gives the unique identifier of each individual, and the corresponding age (in days) and time since infection (in days) values are given in the two other matrices.
(ZIP)
